# Transcriptional control of carbohydrate catabolism by the CcpA protein in the ruminal bacterium *Streptococcus bovis*

**DOI:** 10.1128/aem.00474-23

**Published:** 2023-10-12

**Authors:** Xiujuan Zhao, Ying Zhang, Banglin He, Yu Han, Ben Shen, Yu Zang, Hongrong Wang

**Affiliations:** 1 Laboratory of Metabolic Manipulation of Herbivorous Animal Nutrition, College of Animal Science and Technology, Yangzhou University, Yangzhou, China; Unversidad de los Andes, Bogotá, Colombia

**Keywords:** catabolite control protein A, *Streptococcus bovis*, genome, carbon catabolism, transcriptomic

## Abstract

As a potent, pleiotropic regulatory protein in Gram-positive bacteria, catabolite control protein A (CcpA) mediates the transcriptional control of carbohydrate metabolism in *Streptococcus bovis*, a lactate-producing bacterium that plays an essential role in rumen acidosis in dairy cows. Although the rumen uptake of carbohydrates is multi-substrate, the focus of *S. bovis* research thus far has been on the glucose. With the aid of gene deletion, whole-genome sequencing, and transcriptomics, we have unraveled the role of CcpA in carbohydrate metabolism, on the one hand, and acidosis, on the other, and we show that the *S. bovis* strain S1 encodes “Carbohydrate-Active Enzymes” and that *ccpA* deletion slows the organism’s growth rate and modulates the organic acid fermentation pathways toward lower lactate, higher formate, and acetate in the maltose and cellobiose. Furthermore, this study revealed the different regulatory functions of the CcpA protein in rumen metabolism and acidosis.

**IMPORTANCE:**

This study is important as it illustrates the varying regulatory role of the *Streptococcus bovis* catabolite control protein A protein in carbohydrate metabolism and the onset of acidosis in dairy cattle.

## INTRODUCTION

Rumen acidosis is a common nutritional and metabolic disease in ruminants. In modern intensive production systems, high-concentrate diets are often fed to animals to maximize production performance (1); however, it can lead to rumen acidosis, poor animal health, and economic losses in the livestock industry (2). *Streptococcus bovis*, a Gram-positive bacterium, lactates effectively and plays a key role in the development of rumen acidosis. The metabolism of *Streptococcus bovis* can be modified by ruminal pH (3). Specifically, the lactate dehydrogenase activity of *Streptococcus bovis* increased, and the activity of pyruvate formate lyase decreased when ruminal pH <5.7; thus, the main metabolite of *Streptococcus bovis* is lactate (3). The dissociation constant of lactate is about 10 times stronger than that of volatile fatty acids, so lactate can reduce rumen pH more effectively. *Streptococcus bovis* can switch from mixed acid fermentation to homolactic fermentation when there is excessive substrate and pH is lower than 5.6 (3–5). Although homolactic fermentation generates less ATP than mixed acid fermentation, *S. bovis* has a faster fermentation rate, and it can generate more ATP per hour than any other ruminal bacteria (6).

Studies have demonstrated that, for *Streptococcus bovis*, the catabolite control protein A (CcpA) protein regulates the expression lactate dehydrogenase (LDH), PFL, 6-phosphofructokinase (PFK), pyruvate kinase (PYK), and glyceraldehyde 3-phosphate dehydrogenase that are important in carbohydrate metabolism (7–9). Our lab previously isolated a novel strain of *S. bovis* in goats fed a high concentrate (10). Based on our previous work, we hypothesized that CcpA transcriptionally regulates the fermentation pattern of *S. bovis* S1, which may be affected by carbon source. We focused on the characteristics and functions of the genome, and further studied how the strain adapted to ruminal environment, especially in carbohydrate metabolism. However, the studies on carbohydrate regulation of *Streptococcus bovis* at the transcriptional level are still at the early stage, and most of the work only focus on the gene transcription changes of single coding enzyme, lack of systematic validation of metabolic pathways.

To test this hypothesis, we constructed the *ccpA* gene deletion mutant of *S. bovis* S1 using homologous recombination technology and transcriptomic techniques to investigate the regulatory effects of CcpA protein on carbohydrate metabolism in *Streptococcus bovis* and organic acid fermentation pathways in organisms. In addition, the different regulatory functions of CcpA protein in rumen metabolism and acidosis were also studied.

## RESULTS

### Genome assembly and general features of *S. bovis* S1 genome

**Fig 1:**
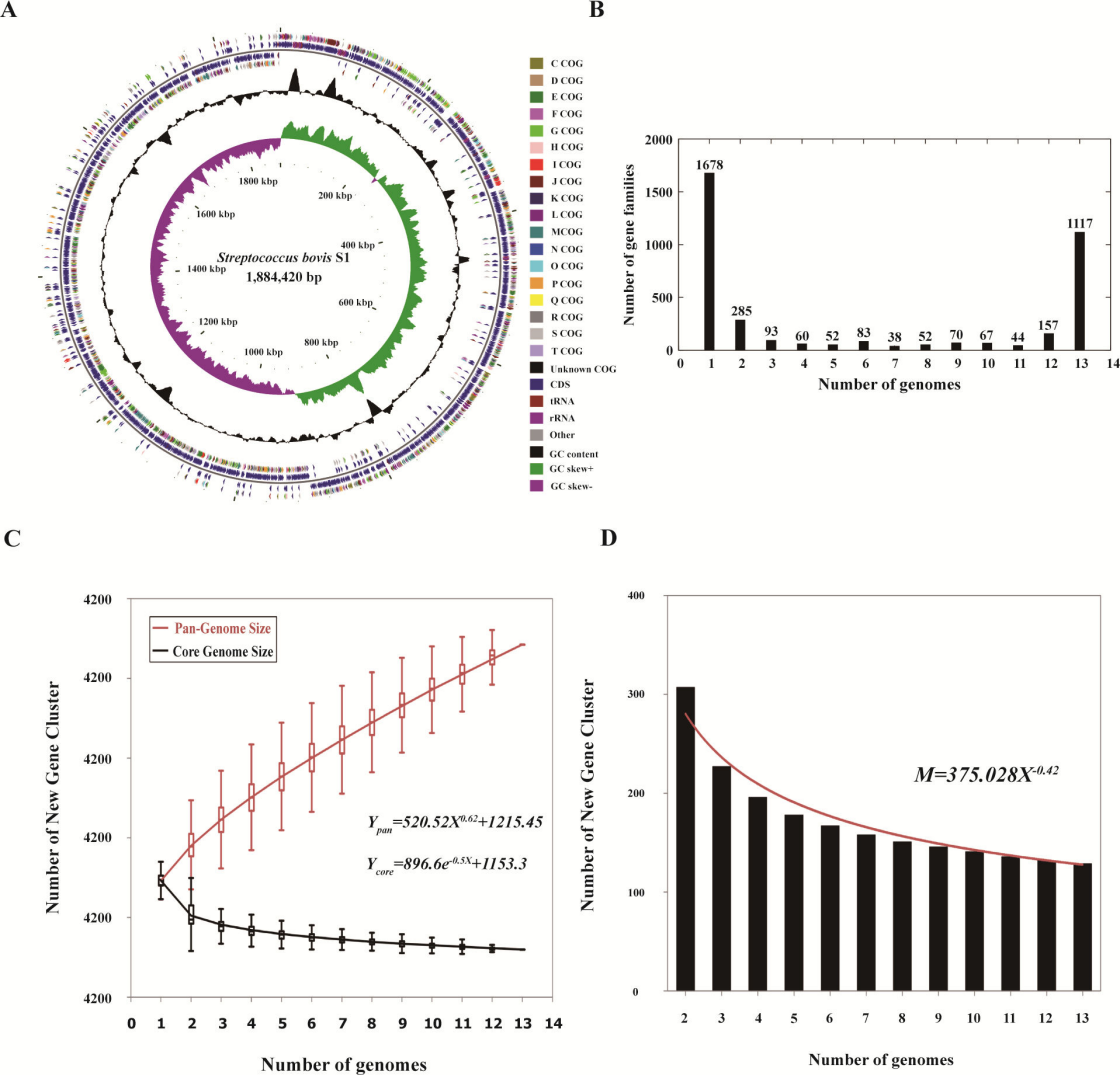
Whole-genome sequencing of *Streptococcus bovis* S1 isolated from goat rumen. (**A**) The circular map of the genome of *Streptococcus bovis* S1. Circles (from inside to out): circle 1 represents the scale; circle 2 shows the GC skew; circle 3 shows the GC content; circles 4 and 7 show the COG (Cluster of Orthologous Groups of proteins) to which each CDS (coding sequence) belongs; and circles 5 and 6 show the positions of CDS, tRNA, and rRNA in the genome. (**B**) The gene family frequency spectrum of *Streptococcus bovis*. (**C–D**) Curves for *Streptococcus bovis* pan-genomes and core genomes. Curves for *Streptococcus bovis* illustrate the number of expected new genes detected with every increase in the number of genomes.

After merging Illumina and PacBio reads, we obtained the complete genome sequence of *S. bovis* S1 (Table 1). It was deposited in GenBank under accession number CP076703. The complete genome for *S. bovis* S1 consisted of a circular 1.88 Mb chromosome (Fig. 1A) with 37.89% cytosine and guanine (GC), which encompassed 1,802 open reading frames (ORFs), 21 rRNAs (7 5S rRNAs, 7 16S rRNAs, 7 23S rRNAs, 69 tRNAs) (Table 2). All ORF lengths accounted for 87.86% of the genome, with 0.956 ORFs per 1,000 bp. The largest ORF length was 6,657 bp, while the average ORF length was 918.78 bp.

**TABLE 1:** Genomic assembly results of *Streptococcus bovis* S1^*a*^

Item	Results
Illumina	
Reads counts	9,490,552
Total base counts	1,423,582,800
G + C content (%)	38.13
Q20 rate (%)	98.24
Q30 rate (%)	94.27
PacBio	
Reads counts	232,108
Total base counts	2,139,984,138
G + C content (%)	38.39
Longest (bp)	118,477
Shortest (bp)	200
N20 length (bp)	13,273
N50 length (bp)	10,315
N90 length (bp)	7,093

^*a*^ Q20 ratio: percentage of bases with a base recognition accuracy of more than 99%; Q30 ratio: the percentage of bases with a base recognition accuracy of 99.9% or more; N20 length: arrange the measured sequences from long to short, and add them in turn. When the obtained length reaches 20% of the total length of the sequence, the length of the last sequence added; N50 length: arrange the measured sequences from long to short, and add them up successively. When the obtained length reaches 50% of the total length of the sequence, the length of the last sequence added; N90 length: arrange the measured sequences from long to short, and add them in turn. When the obtained length reaches 90% of the total length of the sequence, the length of the last sequence added.

**TABLE 2:** Genome-wide characterization of *Streptococcus bovis* S1^*a*^

Item	Features
ORFs	1,802
Genome size (Mb)	1.88
G + C content (%)	37.89
Gene density (per Kb)	0.956
Longest gene length (bp)	6,657
Average gene length (bp)	918.78
Gene length/genome (%)	87.86
The copy number of 5S rRNA	7
The copy number of 16S rRNA	7
The copy number of 23S rRNA	7
The copy number of tRNA	69
Chromosome type	Circular

^*a*^ Circles (from inside to out): circle 1 represents the scale; circle 2 shows the GC skew; circle 3 shows the GC content; circles 4 and 7 show the COG (Cluster of Orthologous Groups of proteins) to which each CDS (coding sequence) belongs; and circles 5 and 6 show the positions of CDS, tRNA, and rRNA in the genome.

### Comparative genomics analysis

Thirteen strains of *Streptococcus bovis* from different sources were used for comparative genomic analysis to explore the evolutionary relationships among the strains. We determined a total number of 3,796 pan-genomic gene families of 13 strains. All the *S. bovis* shared a core genome, consisting of 1,636 orthologs, which was defined as ortholog groups found in 13 strains. The accessory gene families comprised 1,001 dispensable genes (shared by 2 to 12 strains) and 1,678 unique genes (shared by only a single strain) (Fig. 1B).

The pan-genome and core genome sizes of *S. bovis* were calculated using the genomic data. Thirteen random permutations of genomes were carried out to avoid random bias and each genome was added to the pan-genome as a new genome, with the median of the total number of distinct gene families as the size of the pan-genome. A pan-genome curve was plotted by the relationship between the total number of gene families and the number of genomes (Fig. 1C). The power law regression model for the pan-genome curves of *S. bovis* was well described by Heaps’ law mathematical functions and the pan-genome curve formula was *Y*_pan_ = 520.52 *X*^0.62^ + 1215.45 (*R*^2^ = 0.999862). Therefore, the number of genes in the genome of Streptococcus bovis increases with the increase of the number of genomes Similarly, the fitting equation of the relationship between the core genes families and the number of genomes was *Y*_core_ = 896.6e^-0.5X^ + 1153.3 (*R*^2^ = 0.968217), which depicts an extensive shrinking trend in the number of core gene families with the increase of genomes in the curve. According to the formula, the core genome of all the *S. bovis* was estimated to contain about 1,153 genes. Simultaneously, the number of genes corresponding to per additional new genome was plotted in Fig. 1D, and the curve met the following fitting equation: *M* = 375.028 *X*^-0.42^ (*R*^2^ = 0.971134). Thus, we estimated about 124 new genes detected when another genome was added.

**Fig 2:**
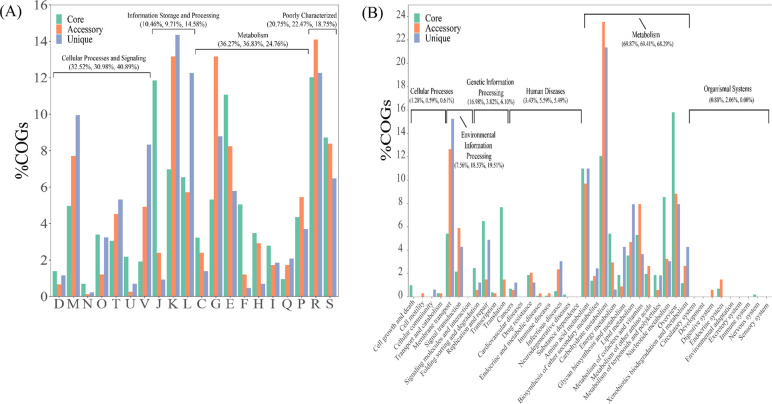
COG and KEGG functional analysis of core genes, accessory genes, and unique genes. (**A**) Based on COG database notes; (**B**) based on KEGG database annotations. D, cell cycle control, cell division, chromosome partitioning; M, cell wall/membrane/envelope biogenesis; N, cell motility; O, posttranslational modification, protein turnover, chaperones; T, signal transduction mechanisms; U, intracellular trafficking, secretion, and vesicular transport; V, defense mechanisms; J, translation, ribosomal structure, and biogenesis; K, transcription; L, replication, recombination, and repair; C, energy production and conversion; G, carbohydrate transport and metabolism; E, amino acid transport and metabolism; F, nucleotide transport and metabolism; H, coenzyme transport and metabolism; I, lipid transport and metabolism; Q, secondary metabolites biosynthesis, transport, and catabolism; P, inorganic ion transport and metabolism; R, general function prediction only; S, function unknown.

In order to explore the function of pan-genome, core genes, accessory genes, and specific genes were annotated by Cluster of Orthologous Groups of proteins (COG) and Kyoto Encyclopedia of Genes and Genomes (KEGG). As shown in Fig. 2A, core genes, accessory genes, and unique genes were enriched in four major functional categories and 20 functional subcategories in the COG annotation database. Further analysis shows that 99.99% of the core genes that are essential to support basic physiological activities have the annotation information of the COG database, in which 36.27% of them were involved in metabolic function. In addition, 32.52% of core genes were involved in information storage and processing. Notably, different from the core genes, more accessory genes were enriched in transcription (13.16%) and carbohydrate transport and metabolism (13.16%). From the perspective of unique genes, they are different from core genes and accessory genes, and they are most abundant in information storage and modification (41.89%). The KEGG pathway analysis was conducted to understand all pan-genomes. As shown in the Fig. 2B, among the core genes, accessory genes, and unique genes, the proportion involved in metabolic pathway is the largest, accounting for 69.87%, 69.41%, and 68.29%, respectively. Among the metabolic pathways, the most enriched genes were carbohydrate metabolism pathway (12.07%, 23.53%, 21.34%), followed by amino acid metabolism (10.99%, 9.71%, 10.98%), and nucleic acid metabolism (8.54%, 3.24%, 3.05%). It can be seen that metabolic capacity is particularly important for *S. bovis*, especially carbohydrate metabolism, which plays an irreplaceable role in the survival and adaptability of various strains.

### Phylogenetic analysis

**Fig 3:**
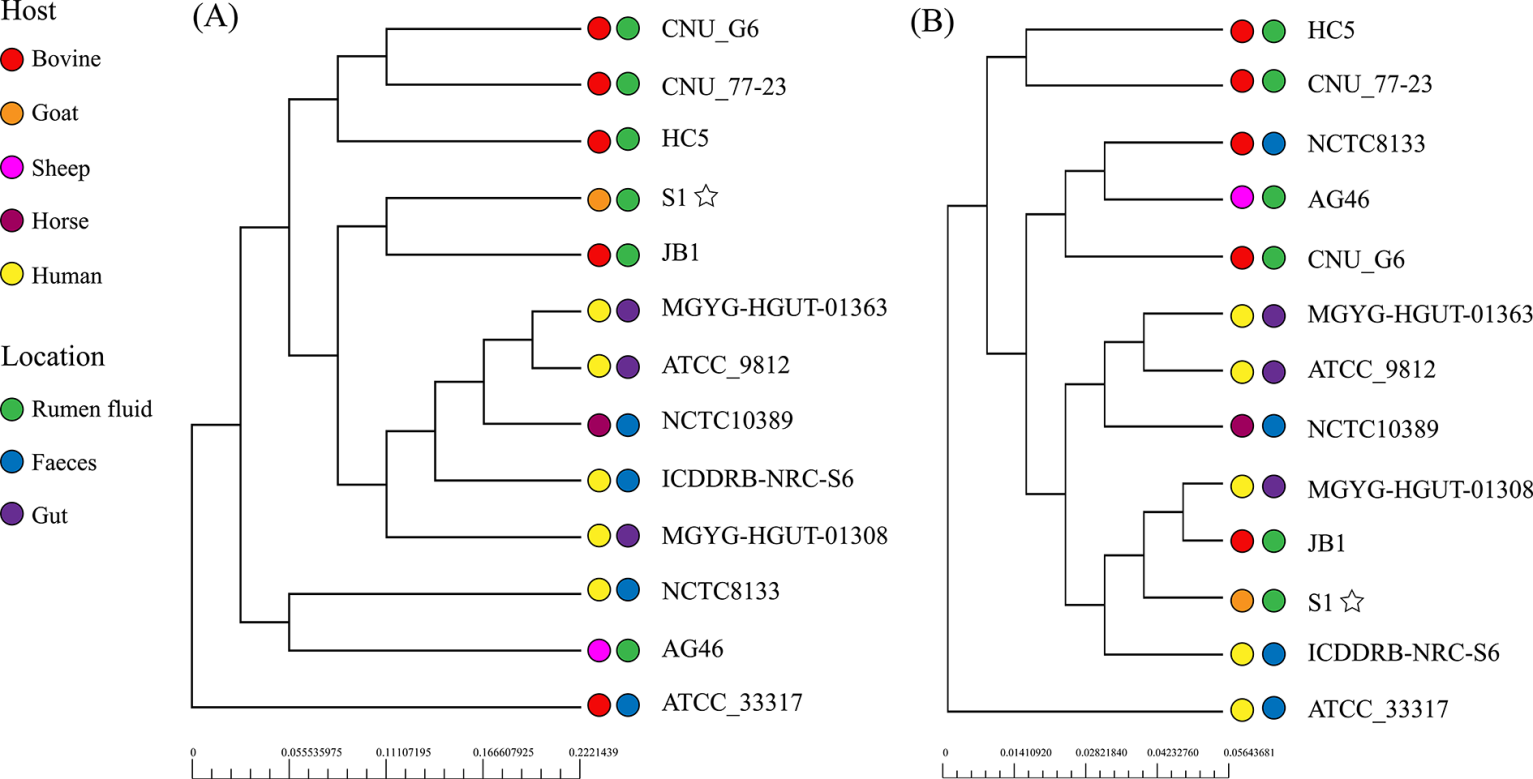
Phylogenetic tree based on pan-core genome of 13 strains of *Streptococcus bovis*. (**A**) Phylogenetic tree analysis based on the pan-genome of 13 *Streptococcus bovis* isolates. (**B**) Phylogenetic relationships based on core gene sets.

We conducted an additional phylogenetic analysis based on the core and pan-genome of the S1 strains and other strains isolated from clear sources (Fig. 2C and D). For the core genome, all strains were grouped into five major phylogroups (Fig. 3A). S1 and JB1 isolated from rumen are closely related, and three strains that were isolated from rumen fluid of bovine including CNU_G6, CNU_77–23, HC5 are classified into the same clade. For the pan-genome, S1 was closely related to JB1 and MGYG-HGUT-01308 followed by ICDDRB-NRC-S6, in which phylogenetic mixing of strains from different isolated sources was observed. Compared with phylogenetic analysis based on pan-genome, strains from the same isolated source have closer phylogenetic relationships and evolutionary relationships are more strongly associated with isolation environment based on core genes, indicating that the core genome of strains from the same isolated source was relatively conservative and performed similar basic functions (Fig. 3B).

### Carbohydrate-active enzymes (CAZymes) analysis

**Fig 4:**
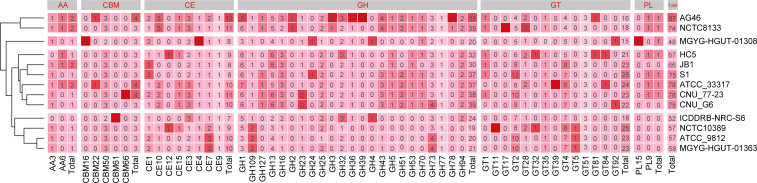
Heatmap of carbohydrate-active enzyme family distribution of 13 strains of *Streptococcus bovis*. AA, auxiliary activities family; CBM, carbohydrate-binding modules family; CE, carbohydrate esterases family; GH, glycoside hydrolases family; GT, glycosyltransferases family; PL, polysaccharide lyases family.

In order to investigate the carbohydrate metabolism and utilization capacity of *S. bovis*, we used the Carbohydrate-Active enzymes (CAZy) database to conduct a comparative analysis of carbohydrate enzymes for 13 strains. As shown in Fig. 4, the 13 strains contained a total of 872 CAZymes, mainly including 16 auxiliary activities, 42 carbohydrate-binding modules, 124 carbohydrate esterases (CEs), 407 glycoside hydrolases (GHs), 276 glycosyltransferases (GTs), and seven polysaccharide lyases. For clarity, the heatmap was built to show the CAZymes distribution of the strains. The number of the CAZymes in different strains was represented by hierarchical clustering, and the 13 strains of *S. bovis* were divided into four branches. Obviously, rumen isolates were on a clade except for AG46, and S1 has a set of the CAZymes similar to JB1. GH family was the most abundant among all strains in the carbohydrate-active enzyme family, accounting for 46.67% of the total. The number of GH1 and GH13 were the most abundant, containing 71 and 61 carbohydrate enzymes, respectively. Compared with the strains isolated from humans, GH1 and GH13 were more abundant in the strain from herbivores, showing an evident association between the host and the presence of GH1 and GH13. The number of GH16 of all the strains was 29, while the CAZymes from the ruminant reached 21, accounting for 72.41%. It was not difficult to see that the strains isolated from ruminants had more abundant GH16 activity. Similarly, both GH5 and GH53 were absent in most strains from human hosts, but were present in ruminant strains. The other GHs were evenly distributed across all strains. In the CE family, S1 and JB1 strains had the largest number of CE1, compared with other strains, but for CE1, CE10, and CE12, JB1 and S1 are absent. Among the GT families, the most abundant was GT2, with 103 CAZymes, especially in strain S1 of this study. These results suggest that the specific or highly enriched carbohydrate-active enzymes in strain S1 may play an important role in its adaptation process.

### Growth curves of *Streptococcus bovis* S1 parental and *ccpA* deletion strains under various carbon source conditions

**Fig 5:**
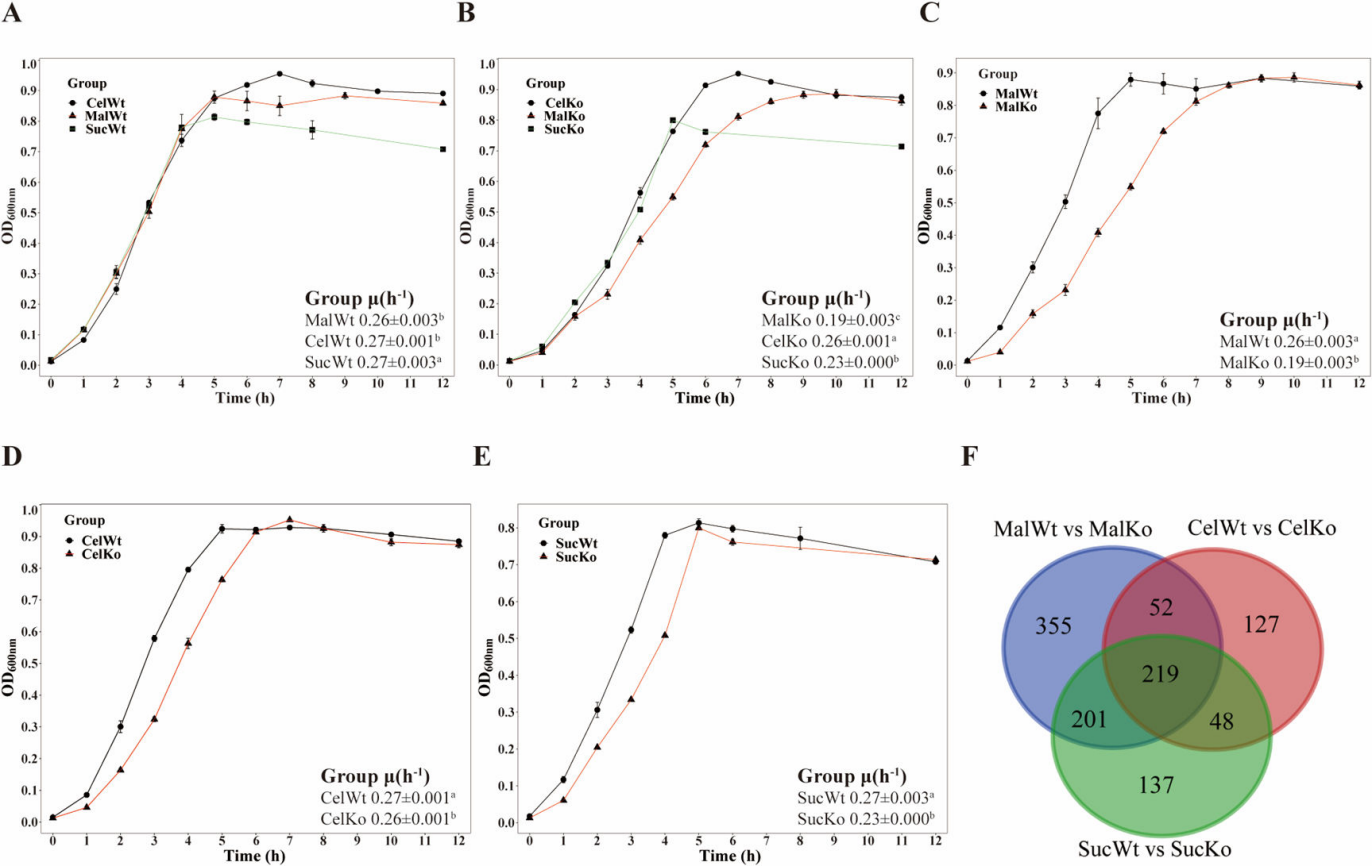
Growth curve and differential genes of parental and *ccpA* deletion S*treptococcus bovis* under various carbon sources. (**A–E**) Growth curves of *Streptococcus bovis* S1 and *ccpA* deletion strain in varied carbon sources. The maximum growth rate data in the figure are presented in the form of “mean ± standard deviation.” Error bars represent standard deviations, and different colors and shapes represent different groups (*P* < 0.05). MalWt, the parental strain grown on maltose; MalKo, the *ccpA* deletion strain grown on maltose; CelWt, the parental strain grown on cellobiose; CelKo, the *ccpA* deletion strain grown cellobiose; SucWt, the parental strain grown on sucrose; SucKo, the *ccpA* deletion strain grown sucrose. (**F–H**) The Venn diagram of transcriptome differentially expressed genes (DEGs) comparison of parental and *ccpA* deletion strains. Black, all DEG counts. Red, upregulated DEG counts. Blue, downregulated DEG counts.

The results show in Fig. 5A that the maximum growth rate of parental strains with sucrose as the carbon source was significantly higher than that of strains with other carbon sources. However, from Fig. 5B, the *ccpA* deletion strain had the highest maximum growth rate among all carbon sources, followed by sucrose and finally maltose. These results indicated that the effect of *ccpA* deletion on *Streptococcus bovis* growth in different carbon sources was different, and the effect on maltose culture was the most significant. It is noteworthy that both parental strain and *ccpA* deletion strains had lower optical density (OD) values upon reaching stationary phase than other carbon sources when sucrose was used as a carbon source, which may be related to the extent of sucrose metabolic utilization by *Streptococcus bovis*. From Fig. 5C through E, the maximum growth rate of *ccpA* deletion strain was significantly lower than that of the parental strain under three carbon sources, respectively.

### Fermentation characteristics and acidogenic pattern of parental strain and *ccpA* deletion strain under various carbon sources

In this study, we investigated the changes of fermentative acid production of parental and *ccpA* deletion strains in various carbon sources at the logarithmic and stationary phases of strain growth (Table 3). In the logarithmic phase, when maltose and cellobiose were used as carbon sources, the *ccpA* deletion strain significantly reduced the lactate production and ratio compared with the parental strain (*P* < 0.05); the concentration and proportion of formate and acetate were increased (*P* < 0.05). But under the condition of sucrose as carbon source, the concentration of lactate was not significant (*P* > 0.05), while the ratio of lactate decreased (*P* < 0.05), and the concentration and ratio of formate and acetate increased (*P* < 0.05). In the acid-producing mode, the deletion strain had lower lactate/formate ratio and lactate/acetate ratio (*P* < 0.05), but the formate/acetate ratio did not change significantly (*P* > 0.05). The results showed that the deletion strain tended to produce more formate and acetate and less lactate in all three carbon sources.

**TABLE 3:** Fermentation characteristics of parental and *ccpA* deletion strain in varied carbon sources^*a*^

Items	Strain	Maltose	Cellobiose	Sucrose
Logarithmic phase				
Lactate (mM）	PA	35.65 ± 0.196^Aa^	34.26 ± 0.089^Ba^	35.41 ± 0.506^Aa^
	CD	32.26 ± 0.999^Ab^	32.42 ± 0.562^Ab^	33.37 ± 1.430^Aa^
Formate (mM）	PA	4.88 ± 0.224^Ab^	4.79 ± 0.100^Ab^	4.38 ± 0.516^Ab^
	CD	7.32 ± 0.247^Ba^	7.87 ± 0.043^Ca^	8.45 ± 0.118^Aa^
Acetate (mM）	PA	2.03 ± 0.202^Ab^	2.20 ± 0.230^Ab^	1.99 ± 0.329^Ab^
	CD	4.00 ± 0.711^Aa^	3.60 ± 0.195^Aa^	4.07 ± 0.124^Aa^
Total acid (mM）	PA	42.57 ± 0.060^Aa^	41.25 ± 0.047^Bb^	41.78 ± 0.644^ABb^
	CD	43.58 ± 1.726^Aa^	44.22 ± 0.948^Aa^	46.25 ± 2.258^Aa^
Lactate ratio (%）	PA	83.76 ± 0.529^Aa^	83.07 ± 0.299^Aa^	84.77 ± 1.66^Aa^
	CD	74.04 ± 1.150^Ab^	74.06 ± 0.224^Ab^	72.18 ± 0.896^Ab^
Formate ratio (%）	PA	11.47 ± 0.511^Ab^	11.61 ± 0.254^Ab^	10.47 ± 1.170^Ab^
	CD	16.80 ± 0.182^Aa^	17.81 ± 0.302^Aa^	18.70 ± 0.246^Aa^
Acetate ratio (%）	PA	4.77 ± 0.476^Ab^	5.32 ± 0.552^Ab^	4.75 ± 0.718^Ab^
	CD	9.16 ± 1.330^Aa^	8.13 ± 0.290^Aa^	9.01 ± 0.584^Aa^
Lactate/formate	PA	7.31 ± 0.346^Aa^	7.16 ± 0.132^Aa^	8.17 ± 1.034^Aa^
	CD	4.41 ± 0.021^Ab^	4.16 ± 0.076^Bb^	3.77 ± 0.228^Cb^
Lactate/acetate	PA	17.67 ± 1.760^Aa^	15.72 ± 0.334^Aa^	18.16 ± 3.261^Aa^
	CD	8.21 ± 1.245^Ab^	9.12 ± 1.652^Ab^	8.37 ± 0.180^Ab^
Formate/acetate	PA	2.42 ± 0.287^Aa^	2.20 ± 0.109^Aa^	2.22 ± 0.278^Aa^
	CD	1.86 ± 0.274^Aa^	2.19 ± 0.270^Aa^	2.08 ± 0.120^Aa^
Stationary phase				
Lactate (mM）	PA	65.67 ± 0.251^Ba^	85.80 ± 2.049^Aa^	51.45 ± 1.010^Ca^
	CD	58.50 ± 1.235^Bb^	75.78 ± 0.091^Ab^	41.05 ± 0.690^Cb^
Formate (mM）	PA	9.58 ± 0.384^Ab^	9.47 ± 0.260^Ab^	7.74 ± 0425^Bb^
	CD	13.75 ± 0.364^Ba^	20.44 ± 0.474^Aa^	11.83 ± 0.758^Ca^
Acetate (mM）	PA	4.98 ± 0.348^Ab^	3.94 ± 0.177^Bb^	3.30 ± 0.279^Bb^
	CD	7.13 ± 0.514^Ba^	10.23 ± 0.208^Aa^	5.80 ± 0.326^Ca^
Total acid (mM）	PA	80.23 ± 0.172^Ba^	99.20 ± 2.468^Ab^	62.49 ± 1.428^Ca^
	CD	79.38 ± 0.687^Ba^	106.44 ± 0.709^Aa^	58.68 ± 1.756^Cb^
Lactate ratio (%）	PA	81.86 ± 0.224^Ba^	86.49 ± 0.088^Aa^	82.35 ± 0.854^Ba^
	CD	73.69 ± 1.140^Ab^	71.19 ± 0.446^Bb^	69.97 ± 0.953^Bb^
Formate ratio (%）	PA	11.94 ± 0.456^Ab^	9.54 ± 0.071^Bb^	12.38 ± 0.507^Ab^
	CD	17.32 ± 0.472^Ba^	19.20 ± 0.320^Aa^	20.15 ± 0.695^Aa^
Acetate ratio (%）	PA	6.21 ± 0.447^Ab^	3.97 ± 0.090^Ca^	5.27 ± 0.356^Bb^
	CD	8.99 ± 0.669^Aa^	9.61 ± 0.131^Ab^	9.89 ± 0.260^Aa^
Lactate/formate	PA	6.86 ± 0.266^Ba^	9.06 ± 0.072^Aa^	6.66 ± 0.335^Ba^
	CD	4.26 ± 0.179^Ab^	3.71 ± 0.084^Bb^	3.48 ± 0.166^Bb^
Lactate/acetate	PA	13.24 ± 0.997^Ca^	21.80 ± 0.146^Aa^	15.67 ± 1.174^Ba^
	CD	8.24 ± 0.732^Ab^	7.41 ± 0.518^ABb^	7.08 ± 0.281^Bb^
Formate/acetate	PA	1.93 ± 0.216^Ba^	2.41 ± 0.065^Aa^	2.35 ± 0.071^Aa^
	CD	1.93 ± 0.103^Aa^	2.00 ± 0.011^Ab^	2.04 ± 0.019^Ab^

^*a*^ Data are presented in the form of “mean ± standard deviation.” PA, parental strain; CD, *ccpA* deletion strain. Superscript uppercase letters A, B, and C represent the changes of acid production of parental or *ccpA* deletion strain under different carbon sources. Superscript lowercase letters a and b represent the changes of acid production of parental and *ccpA* deletion strain under the same carbon source.

### Transcriptome analysis of parental and *ccpA* deletion strains cultured with three carbon sources

From Fig. 5F, the number of genes regulated by CcpA differed under different carbon sources. Under the conditions with maltose as the sole carbon source, a total of 827 genes were changed in the deletion strain compared with the parental strain, with 355 unique differential genes, followed by 605 genes that were significantly changed under the growth conditions with sucrose as the carbon source, with 137 unique differential genes, while under the growth conditions with cellobiose, there were 446 differential genes and 127 unique differential genes. As a result, CcpA has more genes regulated by *Streptococcus bovis* using maltose as a carbon source, which may play a more extensive role in the regulation of *Streptococcus bovis*.

**Fig 6:**
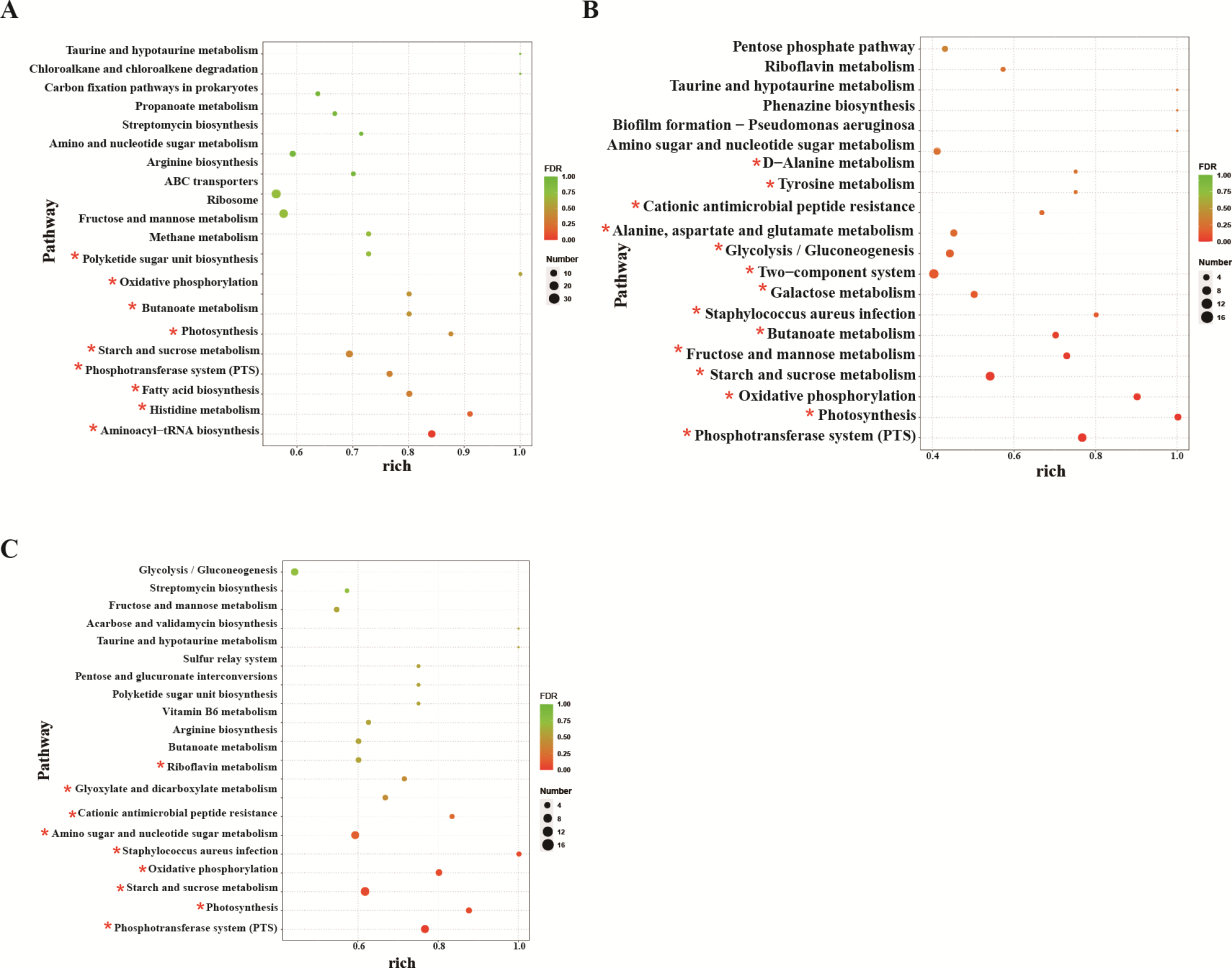
Enrichment analysis of differential genes in parental and *ccpA* deletion strain under various carbon sources. (**A**) The *ccpA* deletion strain grown on maltose versus the parental strain grown on maltose. (**B**) The *ccpA* deletion strain grown on cellobiose versus the parental strain grown on cellobiose. (**C**) The *ccpA* deletion strain grown on sucrose versus the parental strain grown on sucrose. The pathway with asterisk was significantly enriched (*P* < 0.05).

In order to further explore the biological functions of these genes, KEGG pathway enrichment analysis was carried out for the different genes (Fig. 4A through C). The results showed that parental and *ccpA* deletion strains shared regulatory pathways in the three carbon sources, including the phosphotransferase (PTS) system, starch and sucrose metabolism, and oxidative phosphorylation. In addition, under growth conditions in which maltose was the sole carbon source (Fig. 6A), CcpA proteins mainly affected metabolic pathways such as aminoacyl-tRNA biosynthesis, histidine metabolism, fatty acid biosynthesis, butanoate metabolism, and polyketide sugar unit biosynthesis. With cellobiose as a carbon source (Fig. 6B) , in addition to the common metabolic pathways, the deletion of *ccpA* also affected the pathways of fructose and mannose metabolism, butanoate metabolism, galactose metabolism, two-component system, glycolysis/gluconeogenesis, and tyrosine metabolism. Under the condition of using sucrose as carbon source (Fig. 6C), the metabolism of amino sugar and nucleotide sugar metabolism, glyoxylate and dicarboxylate metabolisms, and riboflavin metabolism also changed significantly.

### Identification of differentially expressed genes (DEGs) in different groups by real-time quantitative PCR (RT-qPCR)

**Fig 7:**
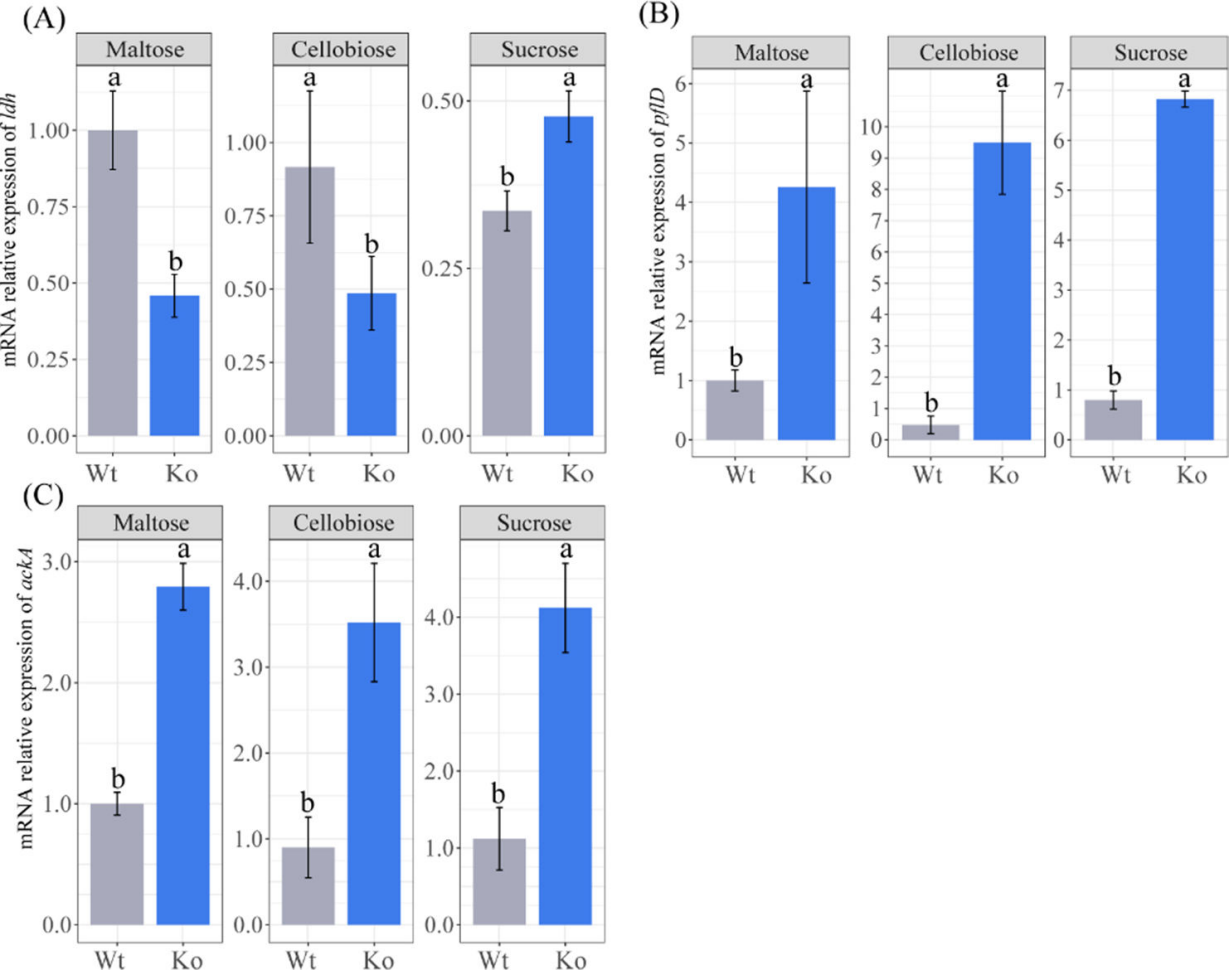
Analysis of *ldh* gene relative expression levels under different carbon sources. Gray bars represent parental strains and blue bars represent missing strain. The error line represents the standard deviation. Lower case letters a and b with different superscripts indicated that there were significant differences in gene expression between parental strain and deletion strain under the same carbon source. *ldh*, L-lactate dehydrogenase; *plf*D, formate C-acetyltransferase; *pfk*A, 6-phosphofructokinase.

To verify the accuracy of transcriptome sequencing data, 16 genes were randomly selected for RT-qPCR quantitative analysis. This study used Pearson correlation analysis to validate data from RT-qPCR and RNA-seq subgroup comparisons (Fig. S1). The results showed that the Pearson correlation coefficient (*R*) of each group was greater than 0.8, and all showed very high significance, which confirmed the reliability and authenticity of the transcriptome sequencing data. In addition, we focused on statistical analysis of *ldh* gene expression (Fig. 7), demonstrating that *ldh* gene expression was downregulated in maltose and cellobiose medium (*P* < 0.05), whereas it was upregulated in sucrose medium (*P* < 0.05).

### Weighted gene co-expression network analysis

**Fig 8:**
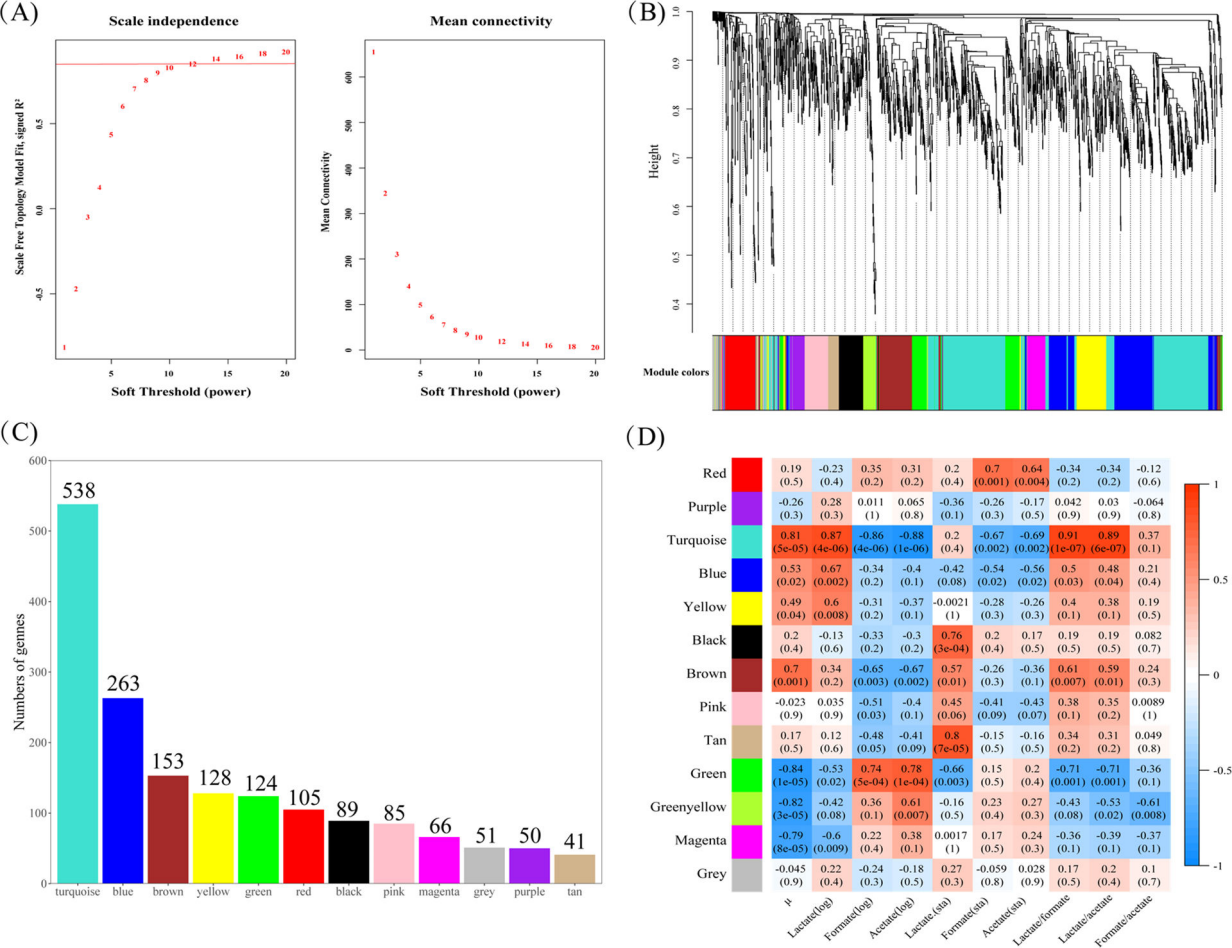
The weighted gene co-expression network analysis based on transcriptome was performed. (**A**) The selection of the soft thresholding power based on scale-free networks. (**B**) The dendrogram displays the different genes that were aggregated into co-expressed modules. (**C**) The number of DEGs to the different modules is shown. (**D**) The relationship between co-expressed modules and the phenotype in both strains. Here, log represents exponential phase and sta represents plateau phase. The top number in each box represents the correlation coefficient and the bottom number represents the *P*-value. Positive correlation is shown in red and negative correlation in blue.

In order to study the regulation of CcpA protein under different carbon sources in *Streptococcus bovis*, we use weighted gene co-expression network analysis (WGCNA) to further mine RNA-seq data. As shown in Fig. 8A, a soft threshold is selected based on the scale-free network distribution. The left panel shows the scale-free simulation, where a larger correlation coefficient is closer to the scale-free network distribution, and the right panel shows the soft threshold β and the average connectivity relationship. It can be seen that a soft threshold of 12 satisfies both the scale-free network distribution and the average connectivity. In Fig. 8B, the dynamic shear method was used to construct co-expression modules, and 12 modules were divided by a soft threshold of β = 6. The number of genes contained in each module is shown in Fig. 8C. From the results, it can be seen that the turquoise module has the highest number of genes, containing 538 genes, followed by more blue module (263), brown module (153), yellow module (128), red module (105), and the least tan module that contains 41 genes.

**Fig 9:**
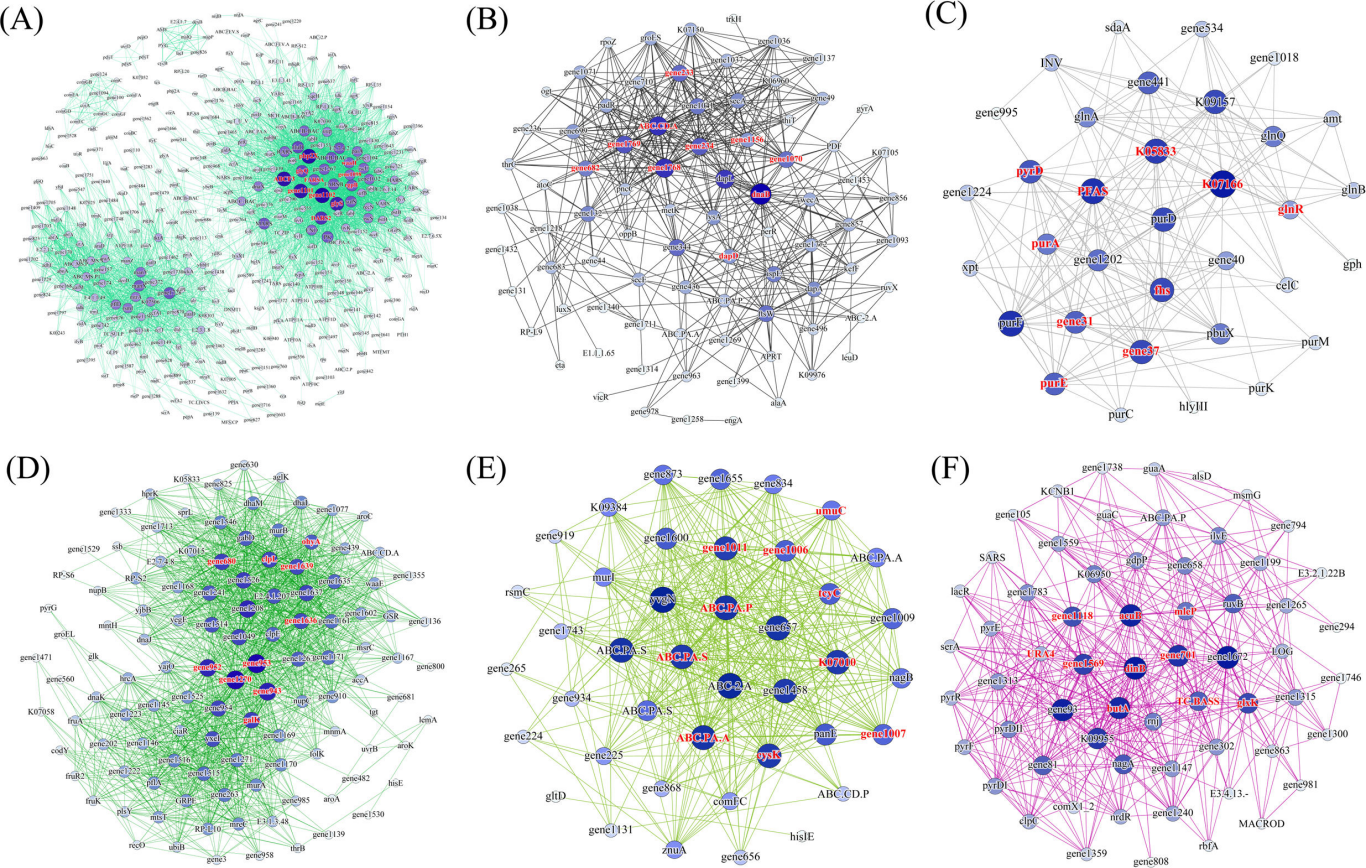
Visualization of gene interaction network of hub genes. (**A**) Gene interaction network of hub genes in turquoise module. (**B**) Gene interaction network of hub genes in black module. (**C**) Gene interaction network of hub genes in tan module. (**D**) Gene interaction network of hub genes in green module. (**E**) Gene interaction network of hub genes in yellowgreen module (**F**) Gene interaction network of hub genes in magenta module. The genes marked in red are hub genes of each module.

In this study, the growth and acid production data of different groups of *Streptococcus bovis* were correlated with each module. Figure 8D is a heatmap of the correlation of 12 modules with each phenotypic index of *Streptococcus bovis*, each color on the left of the graph represents a co-expression module, the numbers in the top row of each box represent specific correlation coefficients, and the values in parentheses represent *P*-values. Based on the obtained data in this study, the correlation coefficient was more than 0.75 and the *P*-value was less than 0.05 as the threshold to judge whether the module was significantly correlated with the phenotype. It can be seen from the results that the turquoise module has a significant correlation with the maximum growth rate of *Streptococcus bovis*, the concentration of lactate, formate, and acetate in the logarithmic phase, the lactate/formate ratio, the lactate/acetate ratio, and the formate/acetate ratio. Meanwhile, black and tan modules were significantly correlated with lactate production in the stationary phase, green modules were correlated with maximal growth rate and logarithmic acetate production, and greenyellow and magenta modules were significantly correlated with maximal growth rate. Next, we analyzed the above-mentioned modules one by one, and used Cytoscape software to find the representative hub genes in each module (Fig. 9A through F; Table 4).

**TABLE 4:** Representative hub genes in significantly related modules^*a*^

Module	Gene	Function
Turquoise modules	*gene1102*	Encoding polysaccharide/phosphoteic acid transporter
	*pbp2X*	Encoding penicillin-binding proteins
	*ABCF3*	Encoding ABC transporter ATP-binding protein
	*glyS*	Encoding glycine-tRNA ligase subunit
	*DARS2*	Encoding aspartic acid-tRNA ligase subunit
	*glyQ*	Encoding glycine-tRNA ligase subunit
	*rgpB*	Encoding rhamnus glycosyltransferase
	*FARSA*	Encoding phenylalanine tRNA synthetase subunit
	*gene1098*	Encoding glycerophosphate transferase
Black module	*gene682*	Encoding MerR family transcription regulator
	*gene233*	Encoding LXG domain of WXG superfamily protein
	*ABC.CD. A*	Encoding a putative ABC transport system ATP-binding protein
	*gene1768*	Encoding an atypical membrane protein
	*dapD*	Encoding 2,3,4,5-tetrahydropyridine-2,6-dicarboxylate anion N-ethyl acyltransferase
	*gene1769*	Encoding a signal peptide protein
	*dnaB*	Encoding a helicase vector
	*gene1156*	Encoding a LytR family transcription regulator
	*gene1070*	Encoding an N-acetyltransferase
Tan module	*K05833*	Encodes ABC transporter ATP-binding protein
	*K07166*	Encodes protein containing ACT domain
	*pyrD*	Encodes dihydrowhey acid oxidase
	*PFAS*	Encodes formylglycine amidine nucleotide synthetase
	*fhs*	Encodes formate tetrahydrofolic acid ligase
	*gene37*	Encodes protein containing DUF523 domain
	*purA*	Encodes adenylate succinate synthase
	*glnR*	Encodes MerR family transcription regulator
	*purE*	Encodes 5-carboxylaminoimidazole nucleotidyl mutagenase
Green module	*gene680*	Encoding halogenase
	*gene1636*	Encoding cellobiose phosphorylase
	*galK*	Encoding galactokinase
	*gene1639*	Encoding glycosyl hydrolase family 16
	*gene943*	Encoding glycosyl cell surface protein hydrolase family 16
	*gene1270*	Encoding airbag protein
	*clpL*	Encoding ATP-binding subunit of ATP-dependent Clp protease ClpL
	*ohyA*	Encoding oleic acid hydratase
Greenyellow module	*ABC.PA. P*	Encodes an amino acid ABC transporter osmotic enzyme
	*cysK*	Encodes L-cysteine synthetase A
	*K07010*	Encodes γ-glutamyl hydrolase
	*ABC.PA. A*	Encoding the amino acid ABC transporter ATP-binding protein
	*ABC.PA.S*	Encoding ABC transporter substrate-binding protein
	*umuC*	Encoding DNA polymerase
	*tcyC*	Encoding ABC transporter and ATP-binding protein
	*gene1011*	Encoding LysR family transcription regulator
	*gene1007*	Encoding ABC transporter membrane protein 1
	*gene1006*	Encoding ABC transporter osmotic enzyme
Magenta module	*gene1569*	Encoding hydrolase
	*butA*	Encoding acetoin reductase
	*dinB*	Encoding DNA polymerase IV
	*acuB*	Encoding acetoin utilization protein
	*gene701*	Encoding uracil DNA glycosylase
	*gene1118*	Encoding FMN-binding protein
	*glxK*	Encoding glycerate kinase
	*mleP*	Encoding malate transporter
	*TC.BASS*	Encoding cotransporter
	*URA4*	Encoding dihydrooratase

^*a*^ The significantly related modules were obtained by correlation analysis between growth and acid production data of different groups of *S. bovis* and each module.

## DISCUSSION

*S. bovis* is a predominant ruminal bacterial species when ruminants are fed a high concentrate diet (1). It is characterized by its strong ability to produce lactate, which contributes to ruminant acidosis, a frequently occurring metabolic disease in ruminant production (5). Therefore, gaining a comprehensive understanding of the biological characteristics of bacterial strains and their regulation of carbohydrate metabolism is helpful for further understanding and alleviating rumen acidosis.

Previous findings have shown that the genomic size of *S. bovis* S1 (1.88 Mb) is similar to that of other strains (1.74 to 1.93 Mb), and the strain S1 shows no significant difference in GC content (37.0% to 37.0%) compared to other sequenced strains (11). This indicates that *S. bovis* has certain environmental adaptability and variable catabolism ability, and does not need to encode a larger array of genes. Additionally, as the number of *S. bovis* strains increases, the size of the pan-genome is also increasing, suggesting abundant genomic diversity between different isolates. (12, 13) Furthermore, regression analysis showed that the bacterial pan-genome of *S. bovis* remains open, as new genes continue to be added to the species gene pool when a new strain is encountered. This result may be explained by the fact that the *S. bovis* is a highly diverse collection of the commensal rumen and gut bacteria, which has a wide spectrum of habitats and functional roles from pathogen to food (14).

Obviously, the match between the clustering relationship and the source of the strain is far from “perfect,” but the phylogenetic trees based on pan-genome shows that most strains cluster according to host. These results align with previous studies indicating that *S. bovis* exhibits a relatively robust and stable genotype in the rumen, regardless of which ruminant species it is isolated from, including sheep, goat, and cattle (15). In contrast to other rumen bacteria, such as *Prevotella ruminicola* (16) and *Butyrivibrio fibrisolvens* (17), *S. bovis* from ruminants appear to be more closely related to each other (18). On the other hand, due to the significant differences in diet between humans and ruminants, as well as variations in microbial community structure (19, 20), specific ecological niches have emerged, promoting the evolution of strains in different directions. As expected, their relationship based on the core genome is less pronounced than those based on the pan-genome. This suggested that genes performing essential functions to support basic physiological activities are similar regardless of the host’s origin.

A preliminary analysis revealed that *S. bovis* possesses the genetic potential to utilize and metabolize a wide range of carbon sources. Specifically, our focus was on the carbohydrate metabolism of *S. bovis*, and the genome data of the strains were further analyzed based on the CAZy database. The results of hierarchical cluster analysis based on enzyme number showed that S1, JB1, CNU_G6, CNU_77–23, and ATCC_33317 were close to each other from ruminants, while ICDDRB-NRC-S6 and MGYG-HGUT-01308 were close to each other from humans. This finding aligns with the results of previous phylogenetic tree analysis, providing further evidence of a correlation between the strain’s genome and its host of isolation. Mainly, in the three CAZy categories, including glycoside hydrolase (GHs), GT, and CE, there were significant differences in the distribution of enzymes among the *S. bovis* strains. GH1, GH13, GH16, GH5, and GH53 were mainly found in herbivores or ruminants, but were absent or rarely present in human strains. The distinct architectural differences in central metabolism between human isolates and those from rumens are likely due to the lack of selective pressure and a limited range of available carbohydrates. These findings support earlier reports on the regulation of gene expression of central metabolic enzymes (21, 22) and the architectural differences (23). Consequently, it is probable that the strains isolated from humans requires fewer such enzymes in their ecological niche due to variations in the range of carbohydrates present in the host. In the rumen, the primary carbon sources are mainly derived from plant fibers (24), which consist predominantly of cellulose, hemicellulose, and lignin. Microorganisms involved in cellulose degradation require three types of enzymes: (i) endoglucanases (ii); exoglucanases, and (iii) β-glucosidases (25, 26). For herbivores or ruminants, GH1 (encoding β-glucosidases) and GH5 (encoding endo-β-1,4-glucanase) play crucial roles in cellulose digestion, while GH16 (encoding xyloglucan:xyloglucosyl transferase) and GH53 (encoding arabinogalactan endo-β-1,4-galactanase) contribute to hemicellulose degradation. In the rumen, *S. bovis* is a typical amylolytic bacterium, which also explains the enrichment of GH13 (encoding α-amylase) in ruminant strains (27, 28). Since strain S1 was isolated from the rumen of goats fed a high concentrate diet, it may require more GT2 (encoding maltodextrin phosphorylase or starch phosphorylase) to degrade the accumulated carbohydrates in the rumen.

The CcpA protein is a vital regulator of carbon metabolism in Gram-positive bacteria with low GC content (29). Previous studies have predominantly focused on glucose as the carbon source to investigate the regulation of CcpA protein, while other carbon sources have received comparatively less attention. However, Chen et al. discovered that the CcpA protein in *Lactobacillus plantarum*, a Gram-positive bacteria, played an equivalent regulatory role in both galactooligosaccharides and glucose as carbon sources (30). These findings suggest that the regulation of CcpA protein may vary depending on the different carbon sources. In this study, we will use homologous recombination to construct *ccpA* deletion strain and parental strain to ferment in different carbon source media, and observe and analyze their growth and key metabolites.

The maximum specific growth rate (μ) is often used to characterize bacterial growth (31). In the growth analysis, *S. bovis* S1 strain exhibited robust growth and metabolic utilization of three carbon sources. It demonstrated the fastest in sucrose medium, while its growth was slower in the cellobiose and maltose medium. These findings differ somewhat from the growth of *Streptococcus bovis* as cultured by Russell et al. (32). This may be due to differences in strains or carbon source concentrations. In the deletion of *ccpA*, the maximum growth rate for each carbon source was significantly lower than that of the parental strain. Similar to the results of this study, some researchers in the study of *Lactococcus lactis* found that after inactivation of CcpA, its growth rate in glucose and sucrose medium decreased by nearly half, while the growth rate in fructose medium decreased by about one-third, even when galactose was used as a carbon source, the strain almost produced growth arrest (33). Additionally, the growth of *Streptococcus mutans ccpA* deletion strain TW1 on glucose, fructose, and mannose medium was significantly slower than that of the parental strain, with the most noticeable difference observed when fructose and mannose were used as carbon sources (34). Thus, the slow growing of *ccpA* deletion strains may be due to inhibition of multiple catabolic pathways, which are typically inhibited by metabolizable carbohydrates during growth. Moreover, it may disturb the balance of alterations of cellular nicotinamide adenine dinucleotide (NAD/NADH) in an unfavorable manner, so as to slow down the growth of the strain (34). However, when Asanuma et al. cultured *Streptococcus bovis* parental strain and *ccpA* deletion strain with glucose and lactose as carbon sources, the growth of the two strains did not change significantly (7). The results indicated that CcpA protein may not affect strain proliferation, potentially due to the difference of carbon source types, and CcpA protein may not play a significant role in all carbon sources.

We also investigated the effect of CcpA protein on the production of organic acids in *Streptococcus bovis* under different carbon sources. Several key metabolites of *Streptococcus bovis*, including lactate, formate, and acetate, were determined and calculated their specific gravity and relative ratios. Lactate is a crucial intermediate in the metabolism of carbohydrates in the rumen and an important driving factor in ruminant acidosis (35). Therefore, the research on lactate metabolism in *Streptococcus bovis* deserves greater attention. Our results indicated that lactate accounted for a significant proportion of all organic acids, which suggest that parental strains primarily underwent lactate-producing homotypic fermentation, which is consistent with previous reports (7, 36). The lactate concentration of parental strain in logarithmic growth period was significantly lower than that of other carbon sources when cellobiose was used as carbon source, indicating that the lactate concentration is also regulated by the carbon source. After reaching the stationary phase, the content and proportion of lactate, formate, and acetate in different carbon sources of parental strains were significantly different, with the most total acid accumulated when cellobiose was used as the carbon source and the least when sucrose was used as the carbon source, which may be due to differences in metabolic pathways of uptake between carbon sources and in the degree of utilization, resulting in changes in metabolic fluxes and thus differences in organic acid production. After deletion of *ccpA* gene, the contents of lactate and acetate in logarithmic phase remained unchanged under different carbon sources, which was consistent with Chen’s results (37). However, the formate content of *ccpA* deletion *Streptococcus bovis* exhibited significantly different among different carbon sources, which may be due to the absence of CcpA magnified the difference in the regulation of formate by carbon sources.

The organic acids of parental and *ccpA* deletion strains also changed obviously in the process of fermentation to the logarithmic phase. The results revealed that the inactivation of CcpA protein significantly decreased the lactate production and ratio, while increasing the formate and acetate production and ratio under the condition that maltose and cellobiose were used as carbon sources. Accordingly, the lactate/formate and the lactate/acetate also decreased significantly, indicating a shift from lactate fermentation to mixed acid fermentation. These findings demonstrate the important role of the CcpA protein in influencing the acid production pattern of *Streptococcus bovis* when maltose and cellobiose are the available carbon sources. In addition, similar changes were observed in strains utilizing sucrose as the carbon source, although lactate production did not change significantly. This suggests that the regulation by CcpA protein is carbon source dependent, and the content of formate is the highest following inactivation of CcpA compared to other carbon sources. This may be the reason why the ratio of organic acids under sucrose conditions has a similar change to that of other carbon sources. Interestingly, a decrease in lactate was observed after the stationary phase of bacteria deletion using sucrose as a carbon source. This is reflected in the difference in OD values between parental and deletion strains after reaching the stationary phase.

The transcriptional regulation of the deletion and parental strains differed under the same carbon source. Enrichment of the phosphoenolpyruvate sugar PTS (PTS system), starch, and sucrose phenotype metabolism was observed under all three carbon sources. Furthermore, *ccpA* deletion can alter fatty acid synthesis and histidine metabolism when maltose and sucrose are used as carbon sources. Fatty acids are the major components of phospholipids in cell membranes, which can maintain membrane stability and normal physiological functions (38), thereby influencing bacterial growth and metabolism. The CcpA protein has been found to regulate fatty acid synthesis indirectly (39). Obviously, in this study, the genes involved in the fatty acid synthesis were inhibited when *ccpA* was deletion and maltose and sucrose were used as carbon sources, thus affecting the growth of the strain, which corresponds to the previous growth curve. Similarly, histidine metabolism was also changed in the presence of maltose and sucrose as carbon sources, indicating that histidine metabolism was also regulated by CcpA and carbon sources.

We conducted an analysis to examine the correlation between the co-expression module constructed by WGCNA and the growth and acid production in various groups, and found that the hub genes of each module were related to carbohydrate metabolism. Interestingly, we found that the hub gene of the turquoise module can also encode various aminoacyl biosynthesis enzymes. Aminoacyl biosynthetase plays an important catalytic role in protein biosynthesis and is involved in many other functions, including regulation of gene expression and amino acid biosynthesis via transamidation pathway (40). These results suggest that these enzymes may affect the growth and acid production of *Streptococcus bovis* by controlling protein synthesis and gene regulation. Within the black module, the hub gene contains two transcription factors, including *gene682* (the MerR protein family encodes the DNA-binding transcription factor AdhR) and *gene1156* (the LytR protein family transcription factor MsrR). It has been demonstrated that MsrR proteins are mainly involved in cell division and septum formation in cells (41). Additionally, the transcription factor *glnR* (encodes a glutamine synthetase inhibitor) is also an important hub gene in the tan module, and *glnR* is a global transcriptional regulator of nitrogen metabolism in Gram-positive bacteria (42, 43). It may play a significant role in nitrogen metabolism of *Streptococcus bovis*. The yellowgreeen module contains a transcription factor, *gene1011* (encodes a LysR family of proteins called YbhD). Its specific function is not yet clear; however, the LysR protein family has been implicated in the regulation of bacterial quorum sensing, bioactivity control, biofilm formation, and cell division, leading to speculation that it may be involved in the regulation of strain growth (44). CcpA protein can regulate carbon metabolism indirectly by regulating the above transcription factors.

### Conclusion

The whole genome analysis revealed the remarkable metabolic capacity of *S. bovis* S1, particularly in terms of carbohydrate metabolism. Furthermore, the deletion of *ccpA* gene inhibits the growth rate of *S. bovis* S1 and regulates the organic acid fermentation pattern toward lower lactate and higher formate and acetate in the maltose and cellobiose. In addition, the pattern of acid production changed from homogenous fermentation to mixed fermentation, but there was no change in lactate production by the sucrose. Moreover, the transcriptomic analysis demonstrated that CcpA protein plays different roles in the regulation of different carbon sources, and participates in the regulation of various carbon metabolic pathways.

## MATERIALS AND METHODS

### Experimental design and sample collection

A strain of *Streptococcus bovis* (*S. bovis* S1, conservation number: CCTCC AB 2016240; GeneBank registry number: CP076703) used in this study was previously isolated and preserved from the rumen fluid of Saanen dairy goats (late lactation) in our laboratory. In this experiment, Man, Rogosa, and Sharpe (MRS) medium was used to enrich *Streptococcus bovis*. Seed bacterial suspensions stored at −80°C were resuscitated overnight on MRS agar plates at 37°C and then cultured in 25 mL serum vials containing 10 mL MRS medium. All procedures are performed in an anaerobic workstation (DG250; Don Whitley Scientific, England). The seed culture was collected for DNA sequencing when the culture attained an OD of 0.55 at 600 nm (at logarithmic phase).

The fermentation medium (Table 5) was prepared according to Asanuma et al. (7). The medium with no carbon source was adjusted to pH 6.5 with 10% sodium hydroxide solution (wt/vol) and sterilized with 118°C high-pressure steam for 15 min. Then, the prepared maltose, cellobiose, and sucrose mother liquor were added to the corresponding medium by a 0.22 µm disposable sterile filtration membrane (Millex-HV, Millipore, USA), respectively. Finally, the fermentation medium with 9 g/L carbon source was obtained. The water used in the experiment was deionized water purified by pure water filter (Millipore, USA).

**TABLE 5:** The composition of fermentation medium^*a*^

Composition	Content (g/L）
K_2_HPO_4_	0.45
KH_2_PO_4_	0.45
(NH4)_2_SO_4_	0.9
NaCl	0.9
CaCl_2_⋅2H_2_O	0.12
MgSO_4_⋅7H_2_O	0.19
Trypsin hydrolyzes casein	1.0
Yeast extract	1.0
H-Cys-OHHCl	0.6
Maltose, cellobiose, or sucrose	9

^*a*^ The maltose, cellobiose, and sucrose mother liquor were added into the culture medium by 0.22 µM one-off aseptic filtration membrane, and the culture medium with carbon source concentration of 9 g/L was obtained.

First, *ccpA*-knockout mutant (*ccpA* deletion) strain of *Streptococcus bovis* was constructed using homologous recombination technology (45). The parental strain and *ccpA* deletion strain of *Streptococcus bovis* were resuscitated and enriched on MRS medium in an anaerobic workstation (DG250; Don Whitley Scientific, England) to make seed culture solution. Second, seed cultures of 1 mL parental strain and its mutant strain in logarithmic growth phase (OD = 0.55) were inoculated into serum vials containing different carbon sources (maltose, cellobiose, or sucrose). The fermentation medium was divided into 250 mL serum bottles; each bottle contained 100 mL fermentation broth. The mouth of the bottle is then sealed and transferred out of the anaerobic workstation (DG250; Don Whitley Scientific, England) and placed in a thermostatic shaker (TS-1102C) at a set temperature of 37°C at 160 rpm/min. During the experiment, a 10% NaOH (wt/vol) was automatically pumped in with an injection pump (TYD02-10; Leadfluid system) to stabilize the medium at a pH of about 6.5. Three replicates were set in each treatment group. Cell growth was monitored by measuring OD values at 600 nm every hour using SpectraMax M5 plate reader (Molecular Devices Corporation, USA). Samples were taken every hour and OD values were measured at a wavelength of 600 nm, which were used to determine acid production relevant indicators of the strain as well as to plot growth curves. In addition, the bacterial suspension was collected when pH was stable at 6.5 and in logarithmic growth period (OD was about 0.65). The collected bacterial suspension was immediately put into liquid nitrogen, quick-frozen, and stored in −80°C refrigerator for subsequent test.

### DNA extraction and genome sequencing

The genomic DNA of *S. bovis* S1 which was grown in MRS medium was extracted and purified using the cetyltrimethylammonium bromide (CTAB) extraction method. DNA quality and integrity were measured using a Qubit Fluorometer (Invitrogen, USA), a Nanodrop Spectrophotometer 2,000 (Thermo Scientific, USA), and agarose gel electrophoresis (Invitrogen, USA). Two whole-genome shotgun libraries were constructed using SMRTbell Template Prep Kit 1.0 (Pacific Biosciences, Menlo Park, CA, USA) and TruSeqTM DNA Sample Prep Kit (Illumina, USA) at Personalbio (Shanghai, China). Then, they were sequenced using the Illumina NovaSeq sequencing (150 bp, paired-end) and the PacBio Sequel platform (standard model), respectively.

### Whole-genome analysis

After the complete genome was obtained, the coding DNA sequences (CDSs) were identified using GeneMarkS (http://topaz.gatech.edu/GeneMark/). tRNA genes and ribosomal RNA genes were predicted using the tRNAscan-SE (http://lowelab.ucsc.edu/tRNAscan-SE/) and Barrnap (http://www.vicbioinformatics.com/software.barrnap.shtml), respectively. Other non-coding RNAs were predicted by comparing the genome sequences to the Rfam database (http://rfam.xfam.org/). The above protein-coding genes were annotated against COG. The genome sequence, the predicted gene, and non-coding RNA were integrated into a GenBank format file, and then, the circular map of the genome was drawn using CGView (http://stothard.afns.ualberta.ca/cgview_server/).

### Comparative genomics analysis

In addition, reference genomes of 12 strains of *S. bovis* for the comparative genome analysis were downloaded from GeneBank database, and the detailed information is shown in Table S1. The whole-genome sequencing files of 12 strains and *S. bovis* S1 were annotated by Prokka software (https://github.com/tseemann/prokka, v1.14.5) to generate GenBank format files.

The results generated in the above steps as input files were analyzed using clustering tools, USEARCH of BPGA software (https://iicb.res.in/bpga/index.html, v1.3), to generate phylogenetic trees, and perform genomic annotations based on the KEGG and COG database.

The curve fitting of the pan-genome growth was performed using a power law regression based on Heap’s law as follows: $Y=A_{pan}X^{B_{pan}}+C_{pan}$ . In addition, core-genome size and number of the genomes meet the following functional relationship: $Y=A_{core}e^{B_{core}X}+C_{core}$ . The fitting was conducted to fit the power law regression by PanGP software (https://pangp.zhaopage.com/manual.html, v1.0.1), where *Y* is the pan-genome size or core-genome size and *X* is the number of the genomes. In addition, the number of new gene families also has the following functional relationship with the number of genomes: $M=aX^{b}$ , where *M* and *X* are the number of new gene families and the number of genomes, respectively.

Genome-level comparisons were made with progressiveMauve using the default parameters (46). Subsequently, carbohydrate-active enzyme annotation was conducted using software hmmscan (http://hmmer.org/) against the CAZy database version 6.0 (http://www.cazy.org/). The results are presented as heatmaps, which were completed by “heatmap” package in R 4.0.1 (https://www.r-project.org/).

### Construction of *ccpA* deletion strain

The knockout strategy is to use the erythromycin-resistant gene as a carrier to the homologous arm with the upstream and downstream homologous sequences of *ccpA*, and then insert the target gene to cause the deletion of *ccpA* gene. It consists of three steps: construction of knockout plasmids, determining the minimum inhibitory concentration of erythromycin, and competent transformation. First, the genomic DNA of *S. bovis* S1 was extracted and used as a template to amplify the upstream and downstream sequences of *ccpA* with primers *ccpA* up1-F/R and *ccpA* down1-F/R (Table 6), respectively. The recovered upstream and downstream sequences (Axygen agarose gel recovery kit) were then linked to blunt vector and transformed into *Escherichia coli* DH5α competent cells at 37°C overnight culture. After the positive clones were identified by PCR, their plasmids (Axygen plasmid miniprep kit) were extracted and sequenced. Then, the deletion primers were designed according to the results of *ccpA* upstream and downstream sequencing. First, the three fragments of *ccpA* upstream and downstream (*ccpA* up2-F-EcoRI/*ccpA* up2-R-SacI and *ccpA* down2-F-BamHI/*ccpA* down2-R-SalI) and erythromycin gene (*Erm*-F-SacI/*Erm*-R BamHI) were amplified, respectively, using pUC19 as plasmid vector and EcoRI/BamHI as double restriction enzyme sites, connect pUC19-*ccpA* up-*Erm,* and transformed into *E.coli* DH5α competent cells. Then, cut *ccpA* down2 and pUC19-*ccpA* up-*Erm* with BamHI/SalI double enzyme*,* respectively, further connection pUC19-*ccpA* up-*Erm-ccpA* down (pCE) and transformed into *E.coli* DH5α competent cells. Take *S. bovis* S1 organism resuspended by 100 µL 10% glycerol, after 15 min of water bath, 5 µg of recombinant carrier pCE was added for electrotransformation (ETF), the parameters of electric shock were set to 2,500V, 25 µF, 200 Ω. Incubate immediately after ETF until *ccpA* deletion strains are screened out, which can be used after PCR and DNA sequencing (Sangon Biotech Co., Ltd., Shanghai) verification.

**TABLE 6:** Primers for construction of *ccpA* deletion strain^*a*^

Primer names	Primer sequences (5´−3´)	Purpose	Length (bp)	Reference or source
*ccpA* up1-F	GTTCCAAGGTCAAACAAAAGTAGAG	Amplify *ccpA* upstream and downstream sequences	1,024	(45)
*ccpA* up1-R	GTAATCGTATCATCAGTGTTCAT			
*ccpA* down1-F	GGTGCGGTAAGTATGCGTATG	Amplify *ccpA* upstream and downstream sequences	1,433	(45)
*ccpA* down1-R	ACGAAATCAATCCACGATAAACA			
*ccpA* up2-F-EcoRI	TGTAAAACGACGGCCAGTGAATTCGTTCCAAGGTCAAACAAAAGTAGAG	Construction of *ccpA*-deletion plasmid	1,053	(47)
*ccpA* up2-R-SacI	AAGCTGTCAAACATGAGAATTAGAGCTCTATTGGACTTCCTTTCTATTTG			
*ccpA* down2-F-BamHI	AGCTTTTGCTAAAGAAGAATTGGATCCTTTCCAAAAAGGATACTATGAC	Construction of *ccpA*-deletion plasmid	1,101	(47)
*ccpA* down2-R-SalI	CAAGCTTGCATGCCTGCAGGTCGACGCAACTTTATCAATGCTACGAC			
*Erm*-F-SacI	CAAATAGAAAGGAAGTCCAATAGAGCTCTAATTCTCATGTTTGACAGCTT	Construction of *ccpA*-deletion plasmid	1,207	(47)
*Erm*-R BamHI	GTCATAGTATCCTTTTTGGAAAGGATCCAATTCTTCTTTAGCAAAAGCT			
*ccpA 1-*F	TGGTGAATCATTACTTGTAAGA	Verification of *ccpA*-deletion plasmid	426	(47)
*ccpA 1-*R	GAGTTCTCTCGCTCACGCACAC			
*ccpA 2-*F	AAACCTTCTTTCTAATTACCCC	Verification of *ccpA*-deletion plasmid	1,827	(47)
*ccpA 2-*R	GGCATAAATCGGCTTGTCAACG			
16 S-F	GAACACCGGTGGCGA	RT-qPCR		(48)
16 S-R	CTCATCGTTTACGGCG			

^*a*^ F, forward primer; R, reverse primer.

### Determination of organic acid content of strain

After collecting and fermenting, the bacterial suspension was centrifuged for 10 min at a rate of 12,000 rpm/min at a constant temperature of 4°C. The supernatant was retained and filtered through a 0.22 µm nylon filter membrane (SCAA-104, ANPEL, China) for sterilization and stored at 4°C.The test used high-performance liquid chromatography (Agilent 2100, USA) to determine the acid production of the strain. The chromatographic conditions are as follows: chromatographic column is a Carbomix H-NP5 column (8% cross-linking degree), detector is a UV detector (UV210 nm), column temperature chamber is set to 55°C, mobile phase is 2.5 mM H_2_SO_4_ solution, flow rate is set to 0.5 mL/min, and injection volume is 10 µL.

### RNA sequencing and transcriptome analysis

In this experiment, bacterial RNA was extracted by TRIzol method. After extraction, the RNA quality was detected. Transcriptome sequencing was performed on Illumina platform using a 2 × 150 bp paired-end configuration. Reads generated by each sample were mapped to the genome of *S. bovis* S1 that we obtained in this study using Bowtie2 with a default parameter (49). The read count was obtained using HTSeq v0.6.1p2 (union mode) and the DEG between the two culture samples was analyzed by DEGseq (50), which converts the raw read count to reads per kilobases per million reads. Fold change >1.5 or <0.67 and a *P*-value <0.05 were defined as the threshold (51). Enrichment analysis was performed using the R software clusterprofile package, and the top 20 paths sorted based on *P-*values were shown.

### Real-time fluorescent quantitative PCR verification

The differentially expressed genes in RNA-seq data were verified by real-time quantitative PCR. The 7,500 Fast Real-Time PCR System (Applied Biosystems, USA) was used for amplification reaction, and a 20 µL reaction system was selected, containing 10 µL 2 × Taq Pro Universal SYBR qPCR Master Mix (Vazyme, China), 0.4 µm of paired positive and negative primers, and 1 µL of cDNA. PCR amplification conditions were as follows: 95°C, 1 cycle for 30 s; 95°C, 10 s, 40 cycles; 60°C, 30 s. The relevant gene expression data were normalized by 2^-ΔΔCt^ method (52), and 16S rDNA was used as internal reference gene. RT-qPCR primer sequences are shown in Table 7.

**TABLE 7:** Primer sequences for RT-qPCR^*a*^

Items	Sequence of primers (5´−3´)	Product size (bp)
*ldh*	F: TTGCTCACGGACTGATTGCTCAAG R: GATTTAGATTCGCTGCTGCATCACG	117
*pflD*	F: CCGTTAAACCAATCCGCGAC R: CCAACCATTCGGCAAGTTCG	114
*ackA*	F: AGTGATGTTCGTGCGGATGT R: CTGAGAACCGAGAGCTGGGAC	113
*fba*	F: TTCCTTGCAGCAGGTATCGG R: TGAACCACCGTGCAATACGA	129
*gene775*	F: CAATTAGCGATTACGGACGGTGTTG R: GTTCAAGGCTGCCAACTTCTTCATC	145
*ndh*	F: CCTGCAATCGTCAAAGGCTTTATCG R: AAGTTGGTGAAGTGGATGTGGTGAG	136
*pck*	F: AAGCCCGTCGAATTTTTGGC R: TGACGACGATGCGCTTGATA	91
*pfkA*	F: GTCACATCCTTCGTGGTGGT R: ACCACGACCTTGTTGAAGCA	98
*pfkB*	F: AGCTTCGATTGGGTCACCTG R: CTGGTGACGGTGCTTTGCTA	149
*pgk*	F: GTCAATGCAGCAAGACCTGG R: AGCTATCAACCTTGGCCGTG	108
*pta*	F: AACCAGGCATTTCCAGAACATCAGG R: TTGCCGTTTCAGCAGTGTTTAAAGC	147
*pyk*	F: TGGGGTGAAAGCCTTGATGT R: CGTTCACCTTGTTCTGCGTG	119
*ldhA*	F: AGCGAATGTCTGGTGTGGATGTTC R: TTCCCGTAGCATTAACCGCAATACC	108
*gene188*	F: AGCCCGCAAGCAACTATGTACTAC R: TGGTGGCACTTTAACGACTGGTC	81
*gene191*	F: AACTCACTTGCTGGTGGACTTCTTC R: AGCGCAGAGTCAGGTTGTTTGTC	104
*pflA*	F: CAGGCGTATCTCGGAACGTA R: TCTGGGGTGAAAATGGTGGG	147

^*a*^ F, forward primer; R, reverse primer.

### WGCNA network co-expression analysis

The read counts expression matrix obtained by sequencing was imported into R software (4.0.01) for format arrangement. First, the R package WGCNA was used to find the appropriate soft thresholding power to satisfy the scale-free network distribution, then the co-expression matrix was constructed by one-step method, and the gene module was visualized by dynamic shear method. Afterward, the correlation between modules and phenotypes was analyzed and a heat map was generated. Subsequently, the relevant modules were selected and imported into the Cytoscape software (3.8.2, Java 11.0.4), and the plugin CytoHubba (v0.1) was used to search for hub genes. Finally, it was imported into the Gephi software (0.9.2) for further visualization.

### Data analysis

The results are expressed in the form of mean ± standard deviation. Analysis of variance or *t*-test procedure was performed with SAS 9.4 software (SAS institute Inc., USA) for statistical analysis, and multiple comparisons were performed by Tukey method, where *P* < 0.05 indicates significant difference. At the same time, based on the logistic function model, R software was used to non-linearly fit the growth curves of different carbon sources and strains of *Streptococcus bovis*. The specific models were as follows (53) :


$$y=y_{0}+\frac{C}{1+e^{\left(4\mu \left(\lambda -t\right)/C+2\right)}}$$

where *y* is the OD value, *y*_0_ is the initial OD value, *t* is the growth time (h), *μ* is the maximum growth rate (h^−1^), *C* is the difference between the maximum and the initial absorbance, and *λ* is the duration of the growth retardation period (h).

## Data Availability

All data generated and/or analyzed during this study are available from the corresponding author upon reasonable request. Genomic sequence of the strain S1 supporting the findings of this study have been deposited in GenBank with the accession code CP076703. The RNA sequences were deposited into the NCBI Sequence Read Archive (SRA) with the accession code SRP323883. This record will be publicly released on 30 June 2024. Meantime, the record is accessible via the temporary link ftp://ftp-trace.ncbi.nlm.nih.gov/sra/review/SRP323883_20230905_121907_f2537af3ef09a82a3b2cc38481ba7a4a. If you require access to this record after this temporary link expires and the record has not been released yet, please email sra@ncbi.nlm.nih.gov for an updated link.

## SUPPLEMENTAL MATERIAL

Supplementary Figure S1 aem.00474-23-s0001.docx Analysis of relative expression levels of differential genes among different groups.

Supplementary Table S1 aem.00474-23-s0002.docx Basic information on streptococcus bovis from different isolated sources.

ASM does not own the copyrights to Supplemental Material that may be linked to, or accessed through, an article. The authors have granted ASM a non-exclusive, world-wide license to publish the Supplemental Material files. Please contact the corresponding author directly for reuse.
