# Supplementary material for: Transcriptional control of carbohydrate catabolism by the CcpA protein in the ruminal bacterium Streptococcus bovis
Source: Appl Environ Microbiol. 2023 Oct 12;89(10):e00474-23. doi: 10.1128/aem.00474-23 (PMC10617382; doi:10.1128/aem.00474-23)
Supplement: Supplementary Figure S1 — Analysis of relative expression levels of differential genes among different groups. [file aem.00474-23-s0001.docx]

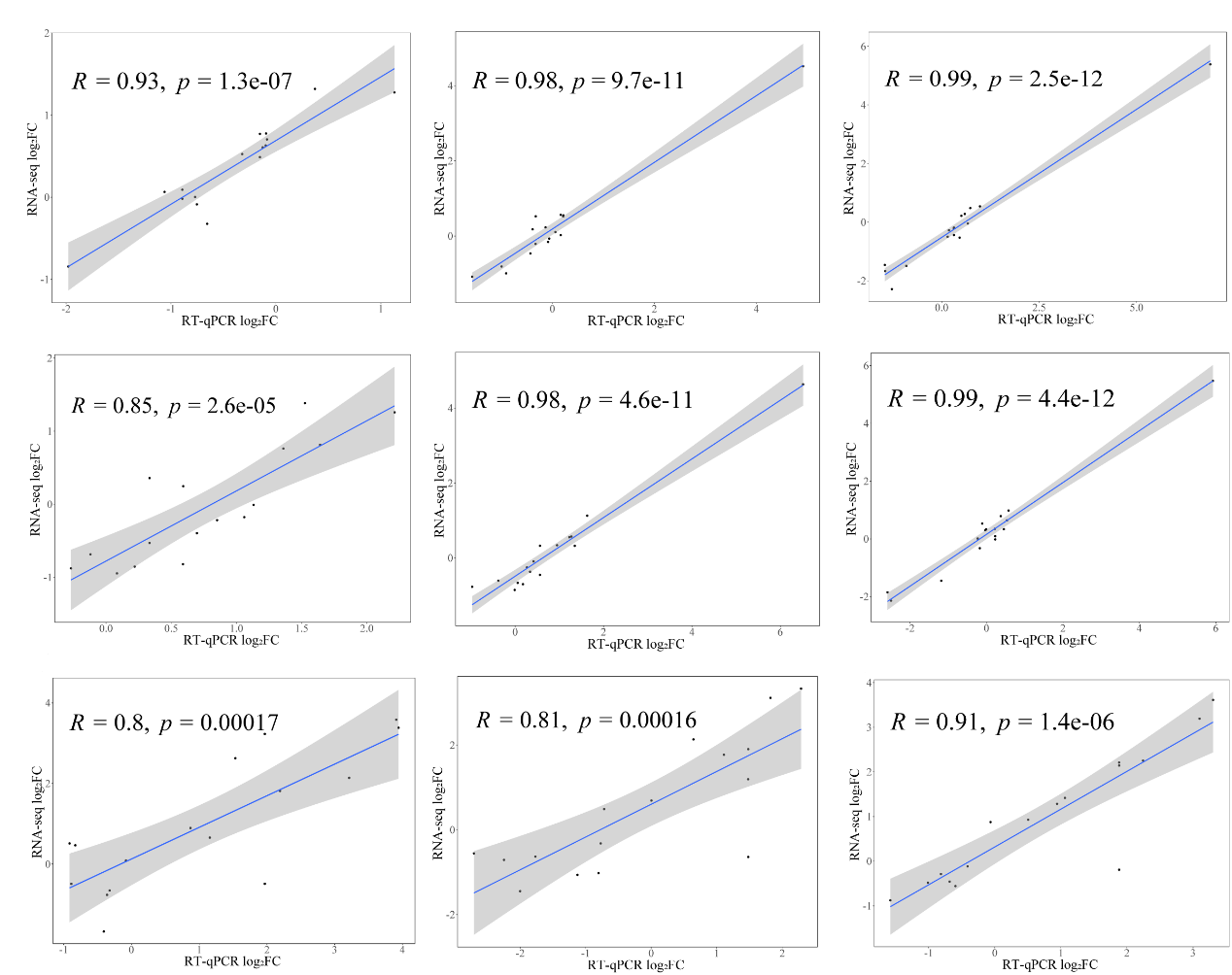


**Supplementary Figure S1** Analysis of relative expression levels of differential genes among different groups. (A) The parental strain grown on cellobiose were compared with those grown on maltose (MalWt vs CelWt); (B) The parental strain grown on sucrose were compared with those grown on maltose (MalWt vs SucWt); (C) The parental strain grown on sucrose were compared with those grown on cellobiose (CelWt vs SucWt); (D) The *ccpA-*deletion strain grown on cellobiose were compared with the *ccpA-* deletion bacteria grown on maltose (MalKo vs CelKo); (E) The *ccpA-*deletion strain grown on sucrose were compared with those grown on maltose (MalKo vs SucKo); (F) The *ccpA-* deletion strain grown on sucrose were compared with the *ccpA-* deletion strain grown on cellobiose (CelKo vs SucKo); (G) The *ccpA-* deletion strain grown on maltose were compared with parental strain grown on maltose (MalWt vs MalKo); (H) The *ccpA-* deletion strain grown on cellobiose were compared with parental strain grown on cellobiose (CelWt vs CelKo); (I) The *ccpA-* deletion strain grown on sucrose were compared with the parental ones grown on sucrose (SucWt vs SucKo).
