# Supplementary material for: Transcriptional control of carbohydrate catabolism by the CcpA protein in the ruminal bacterium Streptococcus bovis
Source: Appl Environ Microbiol. 2023 Oct 12;89(10):e00474-23. doi: 10.1128/aem.00474-23 (PMC10617382; doi:10.1128/aem.00474-23)
Supplement: Supplementary Table S1 — Basic information on streptococcus bovis from different isolated sources. [file aem.00474-23-s0002.docx]

**Supplementary Table S1** Basic information on *streptococcus bovis* from different isolated sources^a^

| **GenBank accession** | **Name** | **rRNA** | **tRNA** | **Total genes** | **Genome size (Mb)** | **G+C content (%)** | **Source** |
| --- | --- | --- | --- | --- | --- | --- | --- |
| S1 | S1 | 21 | 69 | 1802 | 1.88 | 37.9 | Goat rumen |
| GCA_009758075.1 | CNU 77-23 | 21 | 70 | 1880 | 1.91 | 37.4 | Bovine rumen |
| GCF_009758095.1 | CNU G6 | 21 | 70 | 1870 | 1.91 | 37.4 | Bovine rumen |
| GCA_000731085.1 | HC5 | 3 | 31 | 1798 | 1.85 | 37.0 | Bovine rumen |
| GCA_900114525.1 | JB1 | 11 | 31 | 2006 | 1.97 | 37.6 | Bovine rumen |
| GCA_000964315.1 | AG46 | 21 | 70 | 1904 | 1.93 | 37.5 | Sheep rumen |
| GCA_000747195.1 | ATCC 33317 | 3 | 45 | 1801 | 1.84 | 37.2 | Bovine feces |
| GCA_900636565.1 | NCTC10389 | 22 | 62 | 1785 | 1.78 | 37.4 | Horse feces |
| GCA_000187265.1 | ATCC 9812 | 3 | 36 | 1716 | 1.74 | 37.4 | Human gut |
| GCA_902373735.1 | MGYG-HGUT-01308 | 10 | 49 | 1962 | 1.93 | 37.6 | Human gut |
| GCA_902374225.1 | MGYG-HGUT-01363 | 3 | 36 | 1717 | 1.74 | 37.5 | Human gut |
| GCA_001481485.1 | ICDDRB-NRC-S6 | 28 | 89 | 1875 | 1.85 | 37.8 | Children’s stool |
| GCA_901543475.1 | NCTC8133 | 18 | 60 | 1841 | 1.87 | 37.5 | Infant’s feces |

^a^The 12 reference strains included in the analysis were all downloaded from the Genebank database of NCBI, USA
